# Comparative Proteomic Analysis of Tolerant and Sensitive Varieties Reveals That Phenylpropanoid Biosynthesis Contributes to Salt Tolerance in Mulberry

**DOI:** 10.3390/ijms22179402

**Published:** 2021-08-30

**Authors:** Tiantian Gan, Ziwei Lin, Lijun Bao, Tian Hui, Xiaopeng Cui, Yanzhen Huang, Hexin Wang, Chao Su, Feng Jiao, Minjuan Zhang, Yonghua Qian

**Affiliations:** The Sericultural and Silk Research Institute, College of Animal Science and Technology, Northwest A&F University, Yangling 712100, China; gantiantian1987@163.com (T.G.); linxiaoliu510@163.com (Z.L.); baolijun@nwafu.edu.cn (L.B.); ht190322@163.com (T.H.); cuixpssyjs@163.com (X.C.); whyiggy@126.com (Y.H.); wanghexin1111@163.com (H.W.); suchao503@126.com (C.S.); fjiao@nwsuaf.edu.cn (F.J.)

**Keywords:** mulberry, salt stress, TMT proteomics, phenylpropanoid metabolism

## Abstract

Mulberry, an important woody tree, has strong tolerance to environmental stresses, including salinity, drought, and heavy metal stress. However, the current research on mulberry resistance focuses mainly on the selection of resistant resources and the determination of physiological indicators. In order to clarify the molecular mechanism of salt tolerance in mulberry, the physiological changes and proteomic profiles were comprehensively analyzed in salt-tolerant (Jisang3) and salt-sensitive (Guisangyou12) mulberry varieties. After salt treatment, the malondialdehyde (MDA) content and proline content were significantly increased compared to control, and the MDA and proline content in G12 was significantly lower than in Jisang3 under salt stress. The calcium content was significantly reduced in the salt-sensitive mulberry varieties Guisangyou12 (G12), while sodium content was significantly increased in both mulberry varieties. Although the Jisang3 is salt-tolerant, salt stress caused more reductions of photosynthetic rate in Jisang3 than Guisangyou12. Using tandem mass tags (TMT)-based proteomics, the changes of mulberry proteome levels were analyzed in salt-tolerant and salt-sensitive mulberry varieties under salt stress. Combined with GO and KEGG databases, the differentially expressed proteins were significantly enriched in the GO terms of amino acid transport and metabolism and posttranslational modification, protein turnover up-classified in Guisangyou12 while down-classified in Jisang3. Through the comparison of proteomic level, we identified the phenylpropanoid biosynthesis may play an important role in salt tolerance of mulberry. We clarified the molecular mechanism of mulberry salt tolerance, which is of great significance for the selection of excellent candidate genes for saline-alkali soil management and mulberry stress resistance genetic engineering.

## 1. Introduction

Plants need to adapt with the constantly changing environment, including frequent stress environments like drought, salinity, chilling, cold, and heavy metal stress that are not conducive to plant growth and development. In particular, the frequent extreme weather in recent years has caused huge losses to agricultural production [1,2]. Therefore, the understanding of how plants respond to environmental stress is a fundamental question in plant biology [3]. Water deficit and high salinity induced osmotic stress severely restricts plant growth and productivity. Salinity has caused great loss to crop production around the world [4]. The area of saline-alkali land in the world is 950 million hectares, of which the area of saline-alkali land in China is about 99 million hectares. One fifth of arable land is threatened by salinity stress, especially in coastal areas where it is eroded by seawater. Salt stress disrupts many physiological and biochemical processes in plant cell and then induces iron toxicity, osmotic stress, and nutritional deficiencies [5]. Plants evolved to survive in adverse environmental stresses through a variety of strategies, such as developing serious sensors and signaling pathways, stress induced organelles (chloroplasts, mitochondria, peroxisomes, nuclei, and cell walls) response. The stress signals generated from all organelles are integrated to regulate the expression of stress-related genes and other cell activities, thereby restoring cell homeostasis [3]. In addition, a series of physiological processes including the synthesis of osmolytes (e.g., proline, glutathione, mannitol, carbohydrate, glycine betaine, and polyamines) and the activation of antioxidant enzymes (e.g., catalase, glutathione peroxidase, superoxide dismutase, and peroxidase) enhance the tolerance to salt stress [6,7].

Plants have established elaborate mechanisms to cope with salt stress. In recent years, the exploration of salt tolerance mechanisms has been conducted on herbaceous plants like rice, Arabidopsis, wheat and maize, while infrequently in wood plant like cotton and mulberry trees [8,9,10]. High-throughput biological technology such as metabolomics, transcriptomics and proteomics, have been used in the discovery of genetic resources to salt tolerance [11]. The molecular mechanism underlying salt-tolerant rice cultivar (sea rice) has been explored through whole genome sequencing and comparative transcriptome analysis [12]. A comparative transcriptional profiling was performed to explore salt tolerance rice cultivate (FL478) and its sensitive parent (IR29), and more than two thousand genes were found to respond to salt stress [13]. Another comparative transcriptome analysis of salt-tolerant rice under salt stress found that ABA signal transduction is highly related to salt stress [14]. Isobaric tags for relative and absolute quantitation (iTRAQ)-based proteomic analysis of salinity-tolerant rice (*sd58*) and wild-type *Kitaake* revealed that heterotrimeric G protein alpha subunit (RGA1) participates in salt response through ROS scavenging [15]. RNA-seq analysis of cotton found that 14,172 alternative splicing (AS) events were changed under salt stress, and Ser/Arg-rich (SR) proteins related to AS regulation may be crucial in salt stress [16]. Comprehensive RNA sequencing and SWATH-MS-based quantitative proteomics analyses of maize uncovered splicing factors and transcription factors have an important role in salt stress [17].

Mulberry (Rosales: Moraceae), a perennial woody plant, has remarkable nutritional, medicinal value, and economic value, as well as ecological functions. The fruit of mulberry is nutritious for human beings and the leaves are important food for silkworms. Mulberry is also a valuable genetic resource for abiotic stress (e.g., salinity, heavy metal ions, cold and drought) and can be used for drought relief and remediation of polluted water [18]. Genes involved in stress signals were explored in various studies in mulberry. For example, the polyphenol oxidase 1 gene (*MnPPO1*) is regulated during plant stress responses; ABA pathway-related genes *RD29A*, *RD29B*, *RD22*, *ABI3* and *ABI5* were significantly higher in drought tolerance [19]; the receptor for activated C kinase 1 (RACK1) protein is regulated in drought and salt tolerance. DNA methyltransferase genes (*MnCMT2, MnCMT3*, *MnMET1*, *MnDRM1*, and *MnDRM3*) are responsive to biotic stresses [20]. The activities of superoxide dismutase (SOD), catalase (CAT), peroxidase (POD) and ascorbate peroxidase (APX) in mulberry can eliminate the production of oxidative stress induced by Cd stress [21]. MiR397a and its target copper-assisted laccase (LAC) can respond to copper regulatory response in mulberry [22]. Furthermore, its popularization is expected to improve saline-alkali land and gradually turn it into fertile land. Through the release of mulberry (*Morus notablilis*) genome sequence [23], the biological techniques including transcriptome and proteome were used in understanding of the mechanisms of mulberry response to abiotic stress [24,25,26]. However, the comparative proteomic studies on salt tolerance of mulberry are seldom reported. Over the past decade, chemical labeling with isobaric tandem mass tags, such as isobaric tags for relative and absolute quantification reagents (iTRAQ) and tandem mass tag (TMT) reagents, have been employed in a wide range of different studies to explore the signal transduction pathways and protein interaction networks. In this study, we conducted physiological analysis and TMT-based proteomics to elucidate mulberry response to salt stress, which will provide a scientific basis for further understanding of abiotic stress response in mulberry and cultivation of salt-tolerant crops.

## 2. Results

### 2.1. Physiological Responses of Mulberry to Salt Stress

**Figure 1:**
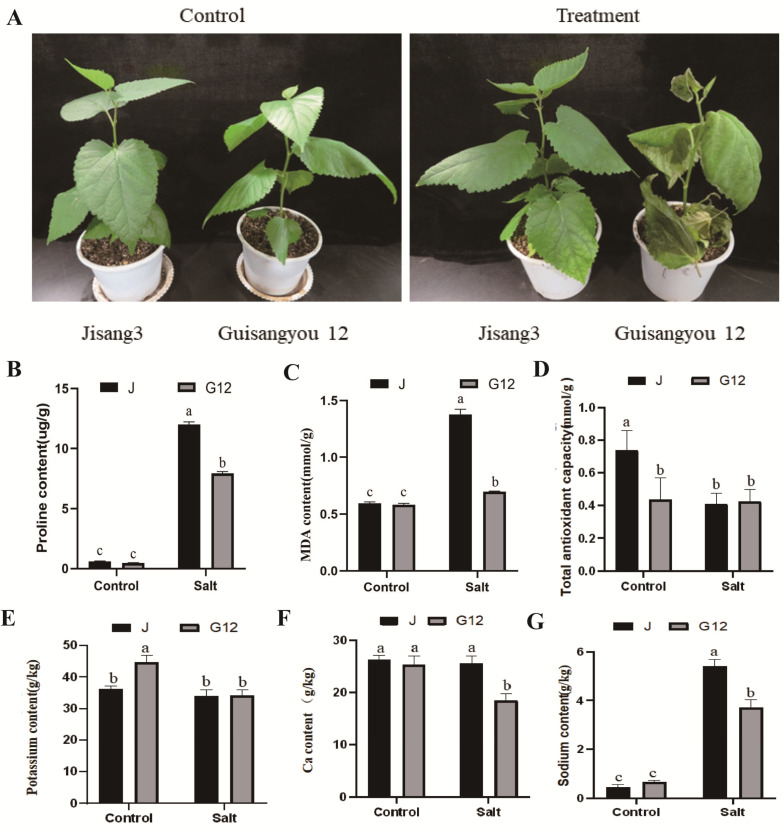
Phenotypic and physiological characteristics of mulberry varieties Jisang3 and Guisangyou12 under control and saline conditions. (**A**) Performance of Jisang3 and Guisangyou12 plants under control and saline conditions. (**B**) Proline content of root in both J and G12 groups. (**C**) MDA content of root in both J and G12 groups. (**D**) Total antioxidant capacity of root of the two varieties in saline condition. (**E**–**G**) Ion content (potassium, calcium, sodium) of root of mulberry varieties Jisang3 and Guisangyou12 under control and saline conditions. Data are means ± SD with three biological replicates. Different letters represent statistically significant differences between control and salt treated plants by Duncan’s multiple range test (*p* < 0.05). Jck, JS, G12ck and G12S represent the two states of Jisang3 and Guisangyou12 under control and salt stress, respectively.

**Figure 2:**
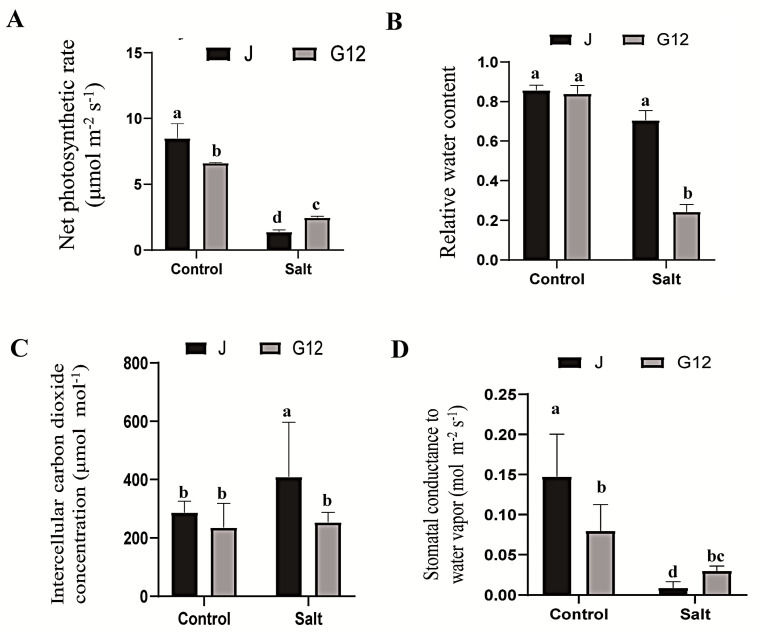
Physiological responses of mulberry to salt stress. (**A**) Net photosynthetic rate of mulberry leaves in both J and G12 groups. (**B**) Relative water content in both J and G12 groups. (**C**) Intercellular carbon dioxide concentration in both J and G12 groups. (**D**) Stomatal conductance to water vapor in both J and G12 groups. Data are means ± SD with three biological replicates. Different letters represent statistically significant differences between control and salt treated plants by Duncan’s multiple range test (*p* < 0.05). Jck, JS, G12ck and G12S represent the two states of Jisang3 and Guisangyou12 under control and salt stress, respectively.

The salt-tolerant mulberry variety Jisang3 and sensitive variety Guisangyou12 were used in this study. After treatment with 200 mM NaCl for 10 days, compared to the Jisang3 seedlings with no obvious stress phenotype, the Guisangyou12 seedlings were wilted and rumpled (Figure 1A). Proline and MDA contents [27] in roots of both varieties were significantly increased after salt stress, while the antioxidant ability of Jisang3 and Guisangyou12 seedlings were significantly decreased (Figure 1B–D). The ion content including potassium, sodium and calcium were also determined. The contents of potassium and calcium were significantly decreased, and the content of sodium was significantly increased (Figure 1E–G). Net photosynthetic rate was further analyzed in Jisang3 and Guisangyou12. It was revealed that the photosynthetic efficiency is significantly reduced in the two varieties during salt stress, but the salt-sensitive variety Guisangyou12 keeps a relatively higher photosynthetic efficiency after salt treatment (Figure 2A). Consistent with plant phenotype, the water content was not decreased in salt-tolerant variety Jisang3 but was significantly reduced in salt-sensitive variety Guisangyou12 (Figure 2B). Intercellular carbon dioxide concentration was significantly increased in Jisang3 but no obvious change in Guisangyou12 (Figure 2C). Stomatal conductance reflecting the degree of stoma opening directly affects the demand for photosynthesis in tree species, and then affects photosynthesis rate. Stomatal conductance has a great influence on the CO_2_ concentration in the intercellular space. As the salt increases, the measured values of stomatal conductance and intercellular concentration decrease sequentially, and the daily change amplitude gradually decreases (Figure 2D). This revealed that the harmony of all varieties is seriously inhibited with the increase of salt stress intensity. These results indicated that the Jisang3 seedlings have a better adaptability to salt stress environment.

### 2.2. Differentially Regulated Proteins (DRPs) in Mulberry Roots

A differential regulated protein (DRP) expressions analysis was performed to determine the responses of mulberry to salt stress and to identify specific strategies of mulberry survival in saline-alkali soil. In total, 1,166,253 mass spectra were produced by mass spectrometer and 162,897 mass spectra were matched with alignment protein, 82,899 peptides in which spectra hits were found and 7044 proteins were detected by spectrum search analysis (Table S1).

In salt-sensitive variety Guisangyou12 after salt stress, there were 255 upregulated DRPs and 532 downregulated DRPs with the fold-change higher than 1.2 (Table 1). With the fold-change higher than 1.5, there were 71 upregulated DRPs and 117 downregulated DRPs in Guisangyou12. These data indicate that a great number of proteins were depressed in salt-sensitive variety by salt stress. In salt-tolerant variety Jisang3, there were 412 upregulated DRPs and 288 downregulated DRPs after salt stress, with the fold-change higher than 1.2 (Table 1). With the fold-change higher than 1.5, there were 68 upregulated DRPs and 64 downregulated DRPs. These data indicate that a large number of proteins were activated in salt-tolerant variety by salt stress. Furthermore, a comparative analysis between Jisang3 and Guisangyou12 showed that 600 DRPs were upregulated and 619 DRPs were downregulated under salt stress, with fold-change higher than 1.2 (Table 1). With the fold-change up to 1.5, there were 169 upregulated DRPs and 165 downregulated DRPs under salt stress.

**Figure 3:**
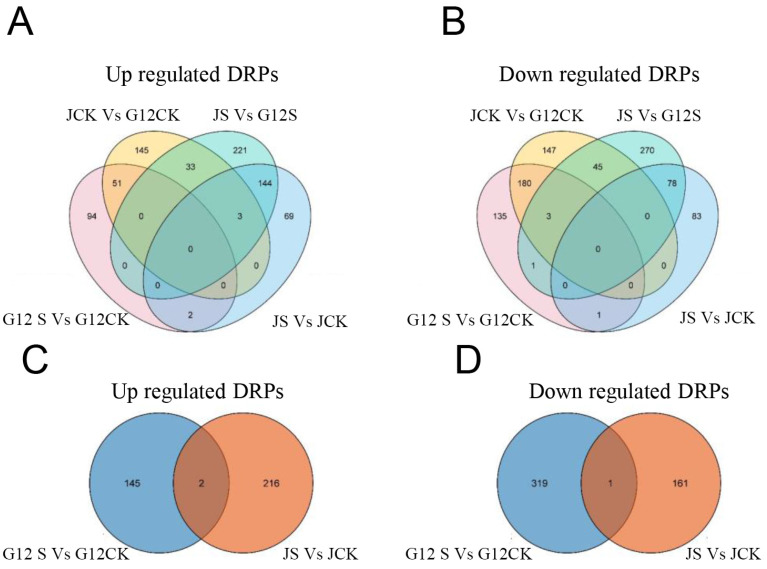
Analysis of Venn diagram. (**A**,**B**) Venn diagram of differentially expressed proteins identified at different treatments. (**C**,**D**) Venn diagram of differentially expressed proteins identified between Jisang3 and Guisangyou12 in the saline condition.

In order to analyze the differential responsive pattern between the salt-sensitive variety and salt-tolerant variety, we created a Venn diagram of the DRPs with fold-change higher than 1.3. As shown in Figure 3, 94 upregulated proteins were unique in G12 group during salt stress and 59 upregulated proteins were unique in G12 group. After salt treatment, 221 proteins were upregulated between two varieties. Among the upregulated proteins, there were only 2 DRPs shared between the two varieties. For the downregulated proteins, there were 135 unique proteins in G12 group and 83 unique proteins in J group, and only one DRP shared between the two varieties. These results indicated that the salt-sensitive mulberry variety and salt-tolerant mulberry variety have different protein profiles in response to salt stress.

**Figure 4:**
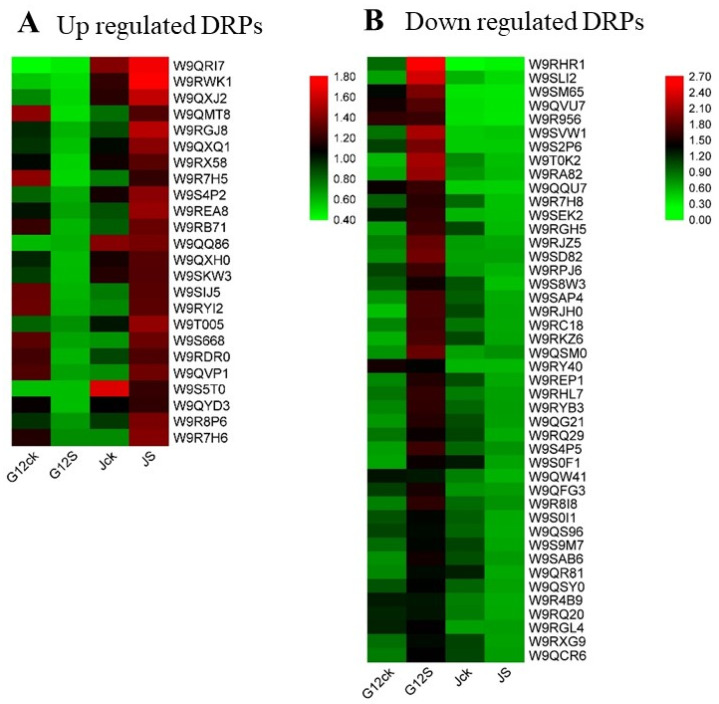
Heatmap of differentially expressed proteins identified in mulberry roots after NaCl treatment. Fold change >1.5. Red represents a high gene expression level and green represents a low gene expression level.

We further selected DRPs with fold-change higher than 1.5 to build a heatmap (Figure 4 and Table S2). Most of them were enzymes that participated in the metabolism. Additionally, these proteins were mostly downregulated in Guisangyou12 but upregulated in Jisang3 after salt stress.

### 2.3. GO Terms and KEGG Pathway in Mulberry Response to Salt Tolerance

To further understand the function of DRPs, these DRPs were enriched in the clusters of orthologous groups of proteins (COG/KOG) category (Figure S2). On the whole, DRPs involved in salt stress were classified into processes including posttranslational modification, protein turnover, chaperones, amino acid transport and metabolism, lipid transport and metabolism, carbohydrate transport and metabolism. The upregulated proteins were classified into processes such as signal transduction mechanisms, intracellular trafficking, secretion, vesicular transport cytoskeleton and cytoskeleton, while the downregulated proteins were classified into processes such as amino acid transport and metabolism, posttranslational modification, protein turnover, chaperones, secondary metabolites biosynthesis, transport and catabolism, lipid transport and metabolism. In Guisangyou12, the upregulated proteins were enriched in the processes including energy production and conversion, posttranslational modification, protein turnover, chaperones, amino acid transport and metabolism, while the downregulated proteins were enriched in the processes including carbohydrate transport and metabolism, intracellular trafficking, secretion, and catabolism. In Jisang3, the upregulated proteins were enriched in processes including signal transduction mechanisms, intracellular trafficking secretion and vesicular transport, cytoskeleton, while downregulated proteins were enriched in processes such as amino acid transport and metabolism, posttranslational modification, protein turnover, chaperones, secondary metabolites biosynthesis transport and catabolism.

To further understand the function of these proteins from a pathway-specific perspective, we subsequently subjected the data to the Kyoto Encyclopedia of Genes and Genomes (KEGG) enrichment (Figure S3). In Guisangyou12, the glucosinolate biosynthesis had significantly upregulated expression under salt stress. The synthesis and degradation of amino acids, beta-alanine metabolism, lysing degradation, valine, leucine and isoleucine degradation and fatty acid degradation were also changed under salt stress. The downregulated DRPs were mostly enriched in phenylpropanoid biosynthesis and steroid biosynthesis. In Jisang3, the upregulated DRPs were enriched in phagosome, pyrimidine and endocytosis, while the downregulated DRPs were enriched in the carbon metabolism, glycolysis/gluconeogenesis and arginine and proline metabolism. In sum, a large number of proteins involved in phenylpropanoid biosynthesis were upregulated while DRPs involved in the carbon metabolism were downregulated. This provides important clues to elucidate the salt tolerant mechanism in mulberry.

Subcellular localization analysis was also performed to understand the localization of the DRPs. As shown in Figure S4, most of the DRPs were located in the nucleus, cytoplasm, plasma membrane and chloroplast, some proteins were located in mitochondria, cytoskeleton, and vacuolar membrane, were also participated in the salt stress response. In the salt-tolerant variety Jisang3, protein transport into nucleus occurred when cytoplasm proteins and chloroplast proteins were degraded. However, in the salt-sensitive variety Guisangyou12, proteins were still processing in cytoplasm and chloroplast, and large amounts of proteins in chloroplast, nucleus and cytoplasm were downregulated. Combined with the physiological and biochemical data (Figure S4), we inferred that DRPs in the salt-tolerant variety were degraded for the synthesis of osmotic stress resistant materials while the salt-sensitive variety could keep a high level of photosynthesis and therefore exhibit salt-stressed phenotype quickly.

### 2.4. Biological Process in Mulberry Response to Salt Stress

After the DRPs in different comparison groups were classified by GO and KEGG pathway, we further performed cluster analysis on these proteins to find the correlation of the DRPs in the comparison groups (Figures S5 and S6). According to the *p*-value of Fisher’s exact test obtained by enrichment analysis, the relevant functions in different groups are grouped together using the hierarchical clustering method and drawn as a heatmap. Different groups of DRPs and color blocks corresponding to the functional description indicate the degree of enrichment. Together with these results, we found some interesting pathways including amino acid synthesis, reactive oxygen scavenging activity and phenylpropanoid biosynthesis, may contribute to salt tolerance. We further analyzed DRPs involved in these pathways, listed in Tables S3–S5. In Guisangyou12, 37 DRPs were involved in amino acid synthesis such as arginine, glycine, serine, valine and tryptophan metabolism and 8 DRPs were involved in glutathione metabolism which were all upregulated, only 10 DRPs related to phenylpropanoid biosynthesis were downregulated during salt stress. In Jisang3, only downregulated DRPs were enriched in these pathways, and 31 DRPs involved in amino acid synthesis and 4 DRPs involved in glutathione metabolism. For comparison of the two varieties after salt stress, 10 DRPs involved in phenylpropanoid biosynthesis were highly upregulated and 90 DRPs involved in amino acid metabolism were downregulated, and another 9 DRPs involved in glutathione metabolism, 14 DRPs involved in peroxisome metabolism and 12 DRPs involved in phenylpropanoid biosynthesis were all downregulated. In a comparison of the two varieties, we found that fatty acid metabolic process (e.g., long-chain fatty acid metabolic process, fatty acid beta-oxidation, fatty acid oxidation, fatty acid catabolic process, monocarboxylic acid catabolic process and carboxylic acid catabolic process) and hyperosmotic salinity response were significantly enriched (Figure S6). Taken together, these results suggest that phenylpropanoid biosynthesis may contribute to salt tolerance in mulberry.

### 2.5. Phenylpropanoid Biosynthesis in Mulberry Response to Salt Stress

**Figure 5:**
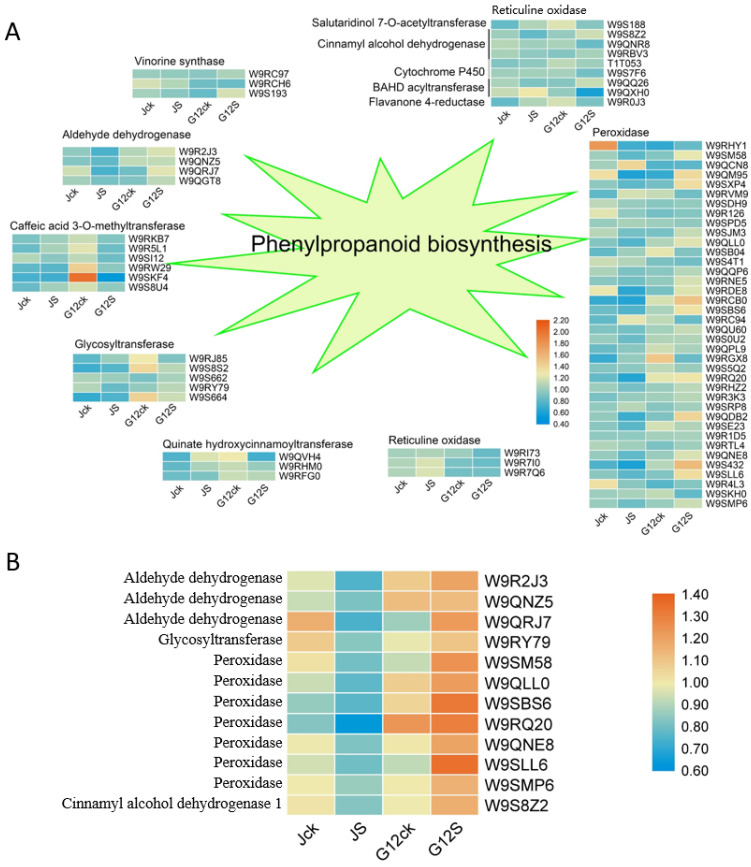
Heatmap of proteins involved in phenylpropanoid biosynthesis pathway. (**A**) Heatmap of proteins identified in phenylpropanoid biosynthesis pathway. (**B**) Heatmap of DRPs involved in phenylpropanoid biosynthesis pathway. Red represents a high gene expression level and blue represents a low gene expression level.

Phenylpropanoids contribute to all aspects of plant responses to biotic and abiotic stimuli [28]. We found that phenylpropanoid biosynthesis pathway is responsive to salt stress (Figure 5A). Aldehyde dehydrogenase were downregulated in Jisang3 and upregulated in Guisangyou12; caffeic acid 3-O-methyltransferase and quinate hydroxycinnamoyl transferase were induced in Jisang3 and depressed in Guisangyou12, while the peroxidase showed a diametrically exposed trend (Figure 5A). The protein abundance of glycosyl transferase was changed irregularly, while reticuline oxidase and vinorine synthase showed a stable expression pattern under salt stress (Figure 5A). As shown in Figure 5B, the DRPs involved in phenylpropanoid biosynthesis were further analyzed. The expressions of aldehyde dehydrogenase, glycosyltransferase, peroxidase, cinnamyl alcohol dehydrogenase were all depressed in Jisang3 but induced in Guisangyou12. Phenylpropanoids including phenol, phenylpropanol, phenylpropionic acid are natural ingredients. Its condensates, leggins, lignans, lignins, and phenylpropanoids are mostly biosynthesized through the shikimic acid pathway [29]. We further measured the contents of coumarin, lignins and flavonoid through high performance liquid chromatography (HPLC). As shown in Figure S7, the coumarin and flavonoid were all induced in Guisangyou12 under salt stress and were correlated with our inference. These results indicated that phenylpropanoids may be involved in salt response and can be used as a target to improve salt resistance in mulberry.

### 2.6. Post-Transcription and Translation Show a Different Pattern in Salt-Tolerant and Salt-Sensitive Mulberry during Early Salt Responsiveness

**Figure 6:**
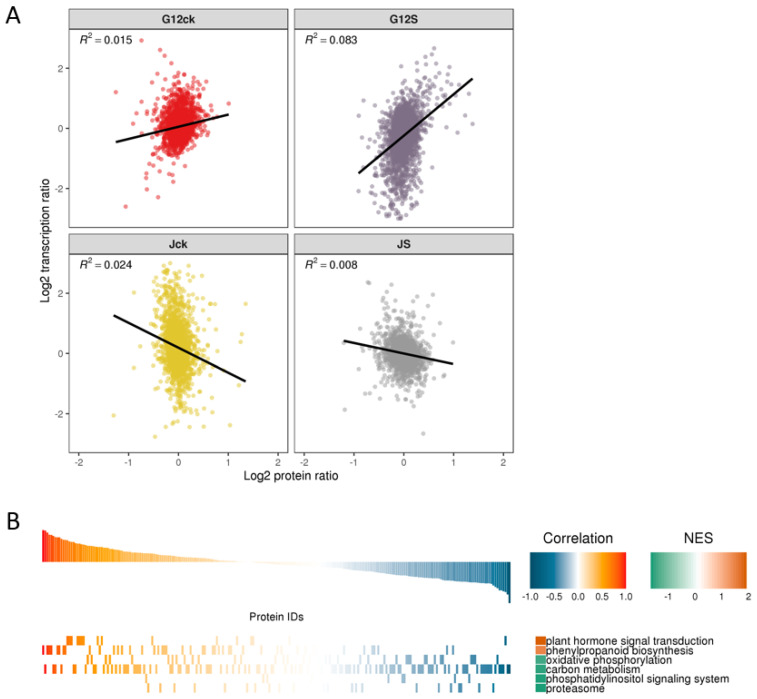
Combined analysis of proteome and transcriptome. (**A**) Scatter plot of transcripts and their corresponding proteins. (**B**) GSEA analysis of KEGG pathway based on quantitative correlation coefficients of transcriptome and proteome.

To further characterize the transcriptome profiles under salt stress, genes involved in salt stress in the two varieties were further screened through RNA-Seq. There were 24,448 genes identified and 4009 genes were both annotated in transcriptome and proteome (Figure S8). The transcriptomic and proteomic data describe the expression of genes at the transcription and translation levels, respectively. In addition to the one-to-one correspondence between transcriptome and proteome, there are other more complex regulatory relationships. Shown in Figure 6A is a scatter plot between the sample’s transcript and its corresponding protein expression. By comparing the quantitative correlation between the two omics, we found that there was a positive regulatory relationship between the protein and the transcript in Guisangyou12 under salt stress while there was a negative regulatory relationship in Jisang3. We further performed the GSEA analysis (Figure 6B) based on KEGG pathway through the above overall analysis of the quantitative correlation between the transcriptome and the proteome. The result showed that there was obvious positive regulatory relationship between the transcriptome and the proteome such as plant hormone signal transduction and phenylpropanoid biosynthesis. Additionally, there were still some negative regulatory relationships between the protein and the transcript such as oxidative phosphorylation, carbon metabolism, phosphatidylinositol signaling system and proteasome. In order to explore the potential relationship between gene transcription level and protein level under multiple experimental conditions, the protein group and transcriptome expression data sets were merged. Then, we used the hclust “ward.D” method to cluster the expression of genes in two dimensions (Figures S9 and S10). The abscissa is the sample in the quantitative study of the proteome and transcriptome, and the ordinate is the protein or transcript quantified in the two omics at the same time. Through the hclust clustering method, these proteins or transcripts were divided into six categories. There is a specific relationship between protein and transcript expression in each category. Preparation of whole transcriptome libraries and deep sequencing were performed by the Annoroad Gene Technology Corporation (Beijing, China).

## 3. Discussion

Soil salinity is a complex environmental issue threat to plant growth and crop production. Mulberry has the characteristics of drought tolerance, salt tolerance, barren tolerance, and strong adaptability to soil [18]. Proteins have a crucial role in stress response and modulate physiological characteristics to develop different phenotypes. In this study, we selected salt-sensitive variety Guisangyou12 and salt-tolerant variety Jisang3 for physiological and proteomic analyses to uncover the mechanism of salt stress adaptability. We found 787 DRPs in Guisangyou12 and 700 DRPs in Jisang3, through the analysis of proteomics data. We found that phenylpropanoid biosynthesis pathway may play important roles in salt tolerance of mulberry. These results may contribute to the genetic improvement of plants for saline-alkali soil management and mulberry stress resistance in genetic engineering.

One important strategy for plants to improve salt tolerance is to re-establish the ionic homeostasis [30]. Stomata are another factor involved in photosynthesis, gas exchange of leaf and microbe interaction [31,32,33]. Studies have also shown that salt stress inhibits the growth and development of mulberry trees [18,34,35]. Proline responds to oxidative processes, protects plants from damage, and acts as a protein stabilizer [36,37,38]. MDA naturally produced form lipid peroxidation and MDA content is used as a parameter to measure the degree of plant cell damage [39,40]. Proline, MDA, were evaluated and upregulated after salt treatment while antioxidant ability reduced in Jisang3 and was unchanged in Guisangyou12. Under salt stress, the content of osmotic adjustment substances such as proline and soluble sugar in mulberry cells increases, which is conducive to its adaptation to the external environment. Ionic homeostasis including potassium, sodium and calcium were detected and sodium content was significantly increased during salt stress. The mulberry Na^+^/H^+^ antiporter NHX1 can have a positive role in salt stress response [41]. Transformation of the *NHX1* gene and the pyrophosphatase gene *AVP1* into Arabidopsis can obtain high salt-tolerant transgenic lines [42]. Net photosynthetic rate was significantly reduced, and the photosynthetic efficiency was higher in the salt-sensitive cultivar (Guisangyou12). Stomata guard gas exchange and enable CO_2_ entry into the leaf for photosynthesis [43,44]. Stomata have a role in water transportation [32,45]. Stomatal conductance balances water–gas exchange and photosynthesis [46]. CO_2_ concentration is also directly related to saline stress and photosynthesis [47]. Stomatal conductance and net photosynthetic rate decreased with the increase of salt content. The intercellular carbon dioxide concentration was increased in Jisang3 while changed little in Guisangyou12. Mulberry trees are salt-tolerant varieties have higher water use efficiency and intercellular CO_2_ concentration than salt-sensitive cultivars [48], but net photosynthetic rate and stomatal conductance of mulberry are lower. Studies have shown that photosynthesis in mulberry trees shows a downregulated trend with the increase of salt concentration [48]. The salt-tolerant mulberry Jisang3 has lower photosynthesis and stomatal conductance, elevates CO_2_ to improve water use efficiency thus avoiding the harm of salt stress. In barley (*Hordeum vulgare*) leaves, elevated CO_2_ partially reduces the impact of salinity on photosynthesis [49]. Comparing the two materials, we found that sodium transfer and ROS scavenge activity were involved in salt stress response. These observations indicate that Jisang3 has a better adaptation to saline conditions than Guisangyou12 plants.

To further elucidate the molecular network response to salt stress between these two varieties, the proteomic data of Jisang3 and Guisangyou12 plants under salt stress was analyzed. We found that 532 of 787 DRPs in Guisangyou12 were downregulated while 412 of 700 DRPs in Jisang3 were upregulated, this indicated that proteins in salt-sensitive plants were depressed while in salt-tolerant plants were induced during salt stress. In Guisangyou12, DRPs were enriched in the glucosinolate biosynthesis, beta-alanine metabolism, lysing degradation, valine, leucine and isoleucine degradation and fatty acid degradation; the detoxification processes such as peroxisome and glutathione metabolism were upregulated in the salt condition. In Jisang3, DRPs related to phagosome, pyrimidine, endocytosis, carbon metabolism, glycolysis/gluconeogenesis and arginine and proline metabolism were enriched after salt treatment. Comparing the two materials, we found that large amounts of proteins involved in much of secondary metabolic pathways were upward expressed including phenylpropanoid biosynthesis, isoflavonoid biosynthesis, cutin, suberine and wax biosynthesis, porphyrin and chlorophyII metabolism, flavonoid biosynthesis, glycerophospholipid metabolism, stilbenoid, diarylheptanoid and gingerol biosynthesis. The pathways such as alanine, aspartate and glutamate metabolism, alpha-linolenic acid metanolism, valine, leucine and isoleucine degradation, 2-oxocarboxylic acid metabolism were enriched in downregulated DRPs. The metabolism related to phenylpropanoid biosynthesis was also marked in Figures S5 and S6. The degradation of fatty acids was in accordance with the increased MDA content. In the previous studies, Luo et al. compared proteomics of two maize sister lines after salt treatment and found that proteins related to phenylpropanoid biosynthesis, phagosome, endocytosis, galactose metabolism, starch and sucrose metabolism, and oxidative phosphorylation were downregulated in the salt sensitive line and oxygen-dependent pentose phosphate pathway, glutathione metabolism and nitrogen metabolism were enhanced [50]. Meng et al. also explored the different responses in sweet potato through high-throughput sequencing in a saline condition and found that ion accumulation, stress signaling, transcriptional regulation, redox reactions, plant hormone signal transduction, and secondary metabolite accumulation may be the response of salt tolerance genotypes [51]. In the analysis of salt treatment to two varieties of *Origanum majorana*, the Tunisian *O. majorana* plants developed tolerance to salinity by improved the process of galactosylation of quercetin into quercetin-3-galactoside and quercetin-3-rhamnoside [52]. Quantitative proteomic analyses of seedling roots from salt-sensitive and salt-tolerant maize were performed by using the iTRAQ method, phenylpropanoid biosynthesis, starch and sucrose metabolism, and the mitogen-activated protein kinase (MAPK) signaling pathway were enriched in salt-tolerant maize while only the nitrogen metabolism pathway was enriched in salt-sensitive maize [53]. Taken together, we found that synthesis and degradation of amino acids, ROS response, carbon metabolism and phenylpropanoid biosynthesis were highly related to salt response. On account of the phenylpropanoids being indicators and key mediators of plant responses to biotic and abiotic stimuli [28,29,54,55], we further analyzed the DRPs involved in phenylpropanoid biosynthesis pathway.

**Figure 7:**
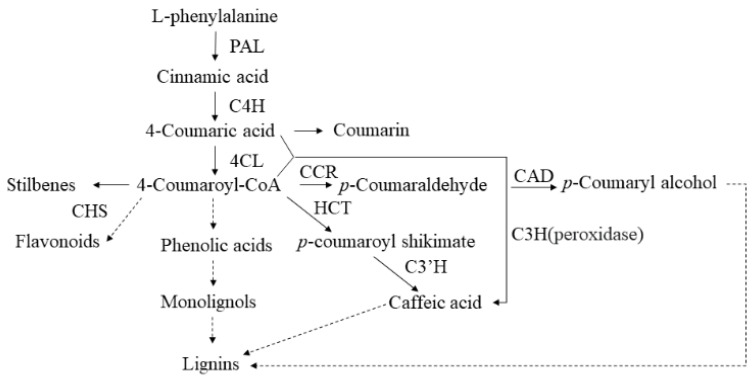
A part scheme of phenylpropanoid metabolism. 4CL, 4-coumarate-CoA ligase; C3H, coumarate 3-hydroxylase; C3′H, p-coumaroyl shikimate 3′ hydroxylase; C4H, cinnamic acid 4-hydroxylase; CAD, cinnamyl alcohol dehydrogenase; HCT, Hydroxycinnamoyl-CoA shikimate/quinate hydroxycinnamoyl transferase; CCR, cinnamoyl-CoA reductase; CHS, chalcone synthase; PAL, phenylalanine ammonia-lyase.

Phenylpropane metabolism is one of the most important plant secondary metabolic pathways, producing more than 8000 metabolites, which play an important role in plant growth and development and plant environmental interaction. Terrestrial plants have evolved a cultivar of branch pathways of phenylpropane metabolism, producing various metabolites such as flavonoids, lignin, lignans, and cinnamic acid amides [50]. Phenylpropane metabolism starts with the phenylalanine produced by the shikimate pathway and undergoes a series of enzymatic reactions to produce secondary metabolites. It is composed of Phenylalanine ammonia-lyase (PAL), Cinnamate 4-hydroxylase (C4H), and 4-coumarate: CoA ligase (4CL) catalyzes to form *p*-coumaric acid: Coenzyme A, which provides precursors for different branches of downstream metabolic pathways (Figure 7). In this study, Aldehyde dehydrogenase was downregulated in Jisang3 and upregulated in Guisangyou12; Caffeic acid 3-*O*-methyltransferase and quinate hydroxycinnamoyltransferase were induced in Jisang3 and degraded in Guisangyou12, while the peroxidase showed a diametrically opposite trend; Glycosyltransferase changes irregularly; Reticuline oxidase and vinorine synthase showed a stable expression pattern under salt stress. Aldehyde dehydrogenase, glycosyltransferase, peroxidase, cinnamyl alcohol dehydrogenase in DRPs were all degraded in Jisang3 and induced in Guisangyou12. Glycosyltransferase including O-glycosyltransferase (OGT) and C-glycosyltransferase (CGT) are related to the production of flavones [56,57]. Cinnamyl alcohol dehydrogenase (CAD) is the final step of monolignol to become caffeyl alcohol, coniferyl alcohol, 5H coniferyl alcohol, and sinapyl alcohol [54]. Peroxidase catalyzes the oxidation of phenylpropanoids to their phenoxyl radicals, and the subsequent nonenzymatic coupling controls the pattern and extent of polymerization [58]. Secondary metabolites explored by HPLC also confirm our conclusion. Abundant metabolites produced from phenylpropanoid biosynthesis pathway coupled with high antioxidant capacity endow mulberry trees with salt tolerance peculiarity.

Comprehensive transcriptome and proteome analyses showed a different expression pattern. A large number of genes showed consistency between the transcript and protein levels in salt-sensitive mulberry Guisangyou12 while salt-tolerant mulberry Jisang3 showed an opposite effect. The integrative transcriptomic and proteomic data are important in deciphering the molecular processes involved in salt stress. Genes and proteins involved in plant hormone signal transduction and phenylpropanoid biosynthesis showed a positive correlation in salt response. Similar to previous studies [53,59,60], the metabolic pathways of plant hormone signal transduction, carotenoid biosynthesis, flavonoid biosynthesis, and starch and sucrose metabolism were involved in salt stress.

## 4. Materials and Method

### 4.1. Plant Material

Two mulberry varieties, Jisang3 (J) and Guisangyou12 (G12) seeds were used in this study. Mulberry seeds were disinfected in 1% HgCl_2_ for 15 min and then soaked in distilled water for 24 h for germination. The mulberry young seedlings were transplanted to sterilized soil substrate for 2 months with a 14 h/10 h (day/night), 25/22 °C (day/night) and 75% air humidity condition. The 21d plants were treated with 200 mM NaCl for two days to impose salinity stress according to the previous study [18,61]. The control group was watered normally. After 10 days, the leaves and roots from each mulberry plant were sampled for physiological analysis and part of them were then stored at −80 °C for TMT-labeled proteomics, respectively.

### 4.2. Physiological Analysis

Photosynthesis of the leaf and the water content was tested. Net photosynthetic rate, intercellular carbon dioxide concentration and stomatal conductance were measured by a portable photosynthesis system (LI-6800; LI-COR, Lincoln, NE, USA). For leaf chamber environment, chamber temperature, relative humidity and CO_2_ concentration were set at 25 °C, 70%, and 400 μmol mol^−1^·s^−1^, each experiment was repeated 10 times. MDA content was analyzed through the TAB method (Banga & Lengyel, 1980) with MDA assay kit (sigma). Proline content, antioxidant capacity and ion content were measured as previously described [62] with test kits (Jiancheng Bioengineering Institute, Nanjing, China). Element content analysis was performed using inductively coupled plasma-optical emission spectrophotometry ICP-OES (ICAP 7000, Thermo Scientific, Waltham, MA, USA).

### 4.3. Protein Extraction, Digestion, and TMT Labeling

Samples of the roots from each mulberry plant were used for protein extraction (n = 3), trypsin digestion and TMT labeling (Thermo Scientific, Waltham, MA, USA). The protein solution was reduced with 5 mM dithiothreitol for 30 min at 56 °C and alkylated with 11 mM iodoacetamide for 15 min at room temperature in darkness. The protein sample was then diluted by adding 100 mM Triethylamonium bicarbonate (TEAB) to urea concentration less than 2 M. Finally, trypsin was added at 1:50 trypsin-to-protein mass ratio for the first digestion overnight and 1:100 trypsin-to-protein mass ratio for a second 4 h digestion. After trypsin digestion, peptide was desalted by Strata X C18 SPE column (Phenomenex) and vacuum dried. Peptide was reconstituted in 0.5 M TEAB and processed according to the manufacturer’s protocol for TMT kit. Briefly, one unit of TMT reagent was thawed and reconstituted in acetonitrile. The peptide mixtures were then incubated for 2 h at room temperature and pooled, desalted and dried by vacuum centrifugation. The tryptic peptides were fractionated into fractions by high pH reverse-phase HPLC using Thermo Betasil C18 column (5 μm particles, 10 mm ID, 250 mm length). The tryptic peptides were treated and subjected to NSI source followed by tandem mass spectrometry (MS/MS) in Q Exactive™ Plus (Thermo Scientific, Waltham, MA, USA) coupled online to the UPLC. A data-dependent procedure alternated between one MS scan followed by 20 MS/MS scans with 15.0 s dynamic exclusion.

### 4.4. Data Processing, Protein Identification, and Quantification

GO Annotation: The Gene Ontology, or GO, is a major bioinformatics initiative to unify the representation of gene and gene product attributes across all species. More specifically, the project aims to maintain and develop its controlled vocabulary of gene and gene product attributes; annotate genes and gene products, and assimilate and disseminate annotation data; provide tools for easy access to all aspects of the data provided by the project. Gene Ontology (GO) annotation proteome was derived from the UniProt-GOA database (http://www.ebi.ac.uk/GOA/ accessed on 20 July 2020). Identified proteins domain functional description were annotated by InterProScan (a sequence analysis application) based on protein sequence alignment method, and the InterPro domain database was used. InterPro (http://www.ebi.ac.uk/interpro/ accessed on 20 July 2020) is a database that integrates diverse information about protein families, domains, and functional sites, and makes it freely available to the public via Web-based interfaces and services. Central to the database are diagnostic models, known as signatures, against which protein sequences can be searched to determine their potential function. InterPro has utility in the large-scale analysis of whole genomes and meta-genomes, as well as in characterizing individual protein sequences.

KEGG Pathway Annotation: KEGG connects known information on molecular interaction networks, such as pathways and complexes (the “Pathway” database), information about genes and proteins generated by genome projects (including the gene database) and information about biochemical compounds and reactions (including compound and reaction databases). These databases are different networks, known as the “protein network”, and the “chemical universe”, respectively. There are efforts in progress to add to the knowledge of KEGG, including information regarding ortholog clusters in the KEGG Orthology database. There, we used wolfpsort, a subcellular localization predication tool, to predict subcellular localization. Wolfpsort is an updated version of PSORT/PSORT II for the prediction of eukaryotic sequences. Special for protokaryon species, subcellular localization prediction tool CELLO was used.

### 4.5. Functional Enrichment

Enrichment of Gene Ontology analysis: Proteins were classified by GO annotation into three categories: biological process, cellular compartment, and molecular function. For each category, a two-tailed Fisher’s exact test was employed to test the enrichment of the differentially expressed protein against all identified proteins. The GO with a corrected *p*-value < 0.05 is considered significant. Enrichment of pathway analysis: Encyclopedia of Genes and Genomes (KEGG) database was used to identify enriched pathways by a two-tailed Fisher’s exact test to test the enrichment of the differentially expressed protein against all identified proteins. The pathway with a corrected *p*-value < 0.05 was considered significant. These pathways were classified into hierarchical categories according to the KEGG website. Enrichment of protein domain analysis: For each category proteins, InterPro (a resource that provides functional analysis of protein sequences by classifying them into families and predicting the presence of domains and important sites) database was researched and a two-tailed Fisher’s exact test was employed to test the enrichment of the differentially expressed protein against all identified proteins. Protein domains with a corrected *p*-value < 0.05 were considered significant.

### 4.6. Enrichment-Based Clustering

For further hierarchical clustering based on differentially expressed protein functional classification (such as GO, Domain, Pathway, Complex). We first collated all the categories obtained after enrichment along with their *p*-values, and then filtered for those categories which were at least enriched in one of the clusters with *p-*value < 0.05. This filtered *p*-value matrix was transformed by the function x = −log10 (*p* value). Finally, these x values were z-transformed for each functional category. These z scores were then clustered by one-way hierarchical clustering (Euclidean distance, average linkage clustering) in Genesis. Cluster membership was visualized by a heat map using the “heatmap.2” function from the “gplots” R-package.

## 5. Conclusions

Collectively, in order to clarify the molecular mechanism of mulberry salt tolerance, comparative proteomic analyses were performed based on the phenotypic, physiological differences in the roots of salt-tolerant and sensitive mulberry varieties after salt treatment. The tolerant and sensitive mulberry genotypes respond differently to salt stress, large amounts DRPs were detected in the two cultivars. Further, our results showed that phenylpropanoid biosynthesis and ROS scavenging system may facilitate the salt tolerance and can be used as the target of genetic breeding. In summary, our results provide a reference for the molecular mechanism for salt condition and reveal the different response pattern between genotypes.

## Figures and Tables

**Table 1:** Differentially expressed protein summary (filtered with threshold value of expression fold change and *p* value < 0.05). Jck, JS, G12ck and G12S represent the two states of Jisang3 and Guisangyou12 under control and salt stress, respectively.

Compare Group	Regulated Type	Fold Change >1.2	Fold Change >1.3	Fold Change >1.5	Fold Change >2
G12S/G12ck	Up-regulated	255	147	71	24
	Down-regulated	532	320	117	15
JS/G12S	Up-regulated	600	402	169	29
	Down-regulated	619	396	165	51
JS/Jck	Up-regulated	412	218	68	11
	Down-regulated	288	162	64	21
Jck/G12ck	Up-regulated	380	232	113	35
	Down-regulated	686	375	135	21

## Data Availability

The data presented in this study are available in the article and supplementary material.

## Supplementary Materials

The following are available online at https://www.mdpi.com/article/10.3390/ijms22179402/s1.

## References

[1] Fedoroff N.V., Battisti D.S., Beachy R.N., Cooper P.J., Fischhoff D.A., Hodges C.N., Knauf V.C., Lobell D., Mazur B.J., Molden D. (2010). Radically rethinking agriculture for the 21st century. Science.

[2] Shabala S., Bose J., Fuglsang A.T., Pottosin I. (2016). On a quest for stress tolerance genes: Membrane transporters in sensing and adapting to hostile soils. J. Exp. Bot..

[3] Zhu J.K. (2016). Abiotic Stress Signaling and Responses in Plants. Cell.

[4] Chaves M.M., Pereira J.S., Maroco J., Rodrigues M.L., Ricardo C.P., Osório M.L., Carvalho I., Faria T., Pinheiro C. (2002). How plants cope with water stress in the field. Photosynthesis and growth. Ann. Bot..

[5] Conde A., Chaves M.M., Gerós H. (2011). Membrane transport, sensing and signaling in plant adaptation to environmental stress. Plant Cell Physiol..

[6] Yamaguchi T., Hamamoto S., Uozumi N. (2013). Sodium transport system in plant cells. Front. Plant Sci..

[7] Deinlein U., Stephan A.B., Horie T., Luo W., Xu G., Schroeder J.I. (2014). Plant salt-tolerance mechanisms. Trends Plant Sci..

[8] Zhao C., Zhang H., Song C., Zhu J.K., Shabala S. (2020). Mechanisms of Plant Responses and Adaptation to Soil Salinity. Innovation.

[9] Wani S.H., Kumar V., Khare T., Guddimalli R., Parveda M., Solymosi K., Suprasanna P., Kavi K.P. (2020). Engineering salinity tolerance in plants: Progress and prospects. Planta.

[10] Sharif I., Aleem S., Farooq J., Rizwan M., Younas A., Sarwar G., Chohan S.M. (2019). Salinity stress in cotton: Effects, mechanism of tolerance and its management strategies. Physiol. Mol. Biol. Plants.

[11] Qin H., Li Y., Huang R. (2020). Advances and challenges in the breeding of salt-tolerant rice. Int. J. Mol. Sci..

[12] Chen R., Cheng Y., Han S., Van Handel B., Dong L., Li X., Xie X. (2017). Whole genome sequencing and comparative transcriptome analysis of a novel seawater adapted, salt-resistant rice cultivar-sea rice 86. BMC Genom..

[13] Mirdar M.R., Shobbar Z.S., Babaeian J.N., Ghaffari M.R., Nematzadeh G.A., Asari S. (2019). Dissecting molecular mechanisms underlying salt tolerance in rice: A comparative transcriptional profiling of the contrasting genotypes. Rice (N.Y.).

[14] Sun B.R., Fu C.Y., Fan Z.L., Chen Y., Chen W.F., Zhang J., Jiang L.Q., Lv S., Pan D.J., Li C. (2019). Genomic and transcriptomic analysis reveal molecular basis of salinity tolerance in a novel strong salt-tolerant rice landrace Changmaogu. Rice (N.Y.).

[15] Peng P., Gao Y., Li Z., Yu Y., Qin H., Guo Y., Huang R., Wang J. (2019). Proteomic analysis of a rice mutant sd58 possessing a novel d1 allele of heterotrimeric G protein alpha subunit (RGA1) in salt stress with a focus on ROS scavenging. Int. J. Mol. Sci..

[16] Zhu G., Li W., Zhang F., Guo W. (2018). RNA-seq analysis reveals alternative splicing under salt stress in cotton, Gossypium davidsonii. BMC Genom..

[17] Chen M., Lu C., Sun P., Nie Y., Tian Y., Hu Q., Das D., Hou X., Gao B., Chen X. (2021). Comprehensive transcriptome and proteome analyses reveal a novel sodium chloride responsive gene network in maize seed tissues during germination. Plant Cell Environ..

[18] Liu Y., Ji D., Turgeon R., Chen J., Lin T., Huang J., Luo J., Zhu Y., Zhang C., Lv Z. (2019). Physiological and proteomic responses of Mulberry trees (*Morus alba* L.) to combined salt and drought stress. Int. J. Mol. Sci..

[19] Li R., Hu F., Li B., Zhang Y., Chen M., Fan T., Wang T. (2020). Whole genome bisulfite sequencing methylome analysis of mulberry (*Morus alba*) reveals epigenome modifications in response to drought stress. Sci. Rep.-UK.

[20] Xin Y., Ma B., Zeng Q., He W., Qin M., He N. (2021). Dynamic changes in transposable element and gene methylation in mulberry (*Morus notabilis*) in response to Botrytis cinerea. Hortic. Res..

[21] Guo Z., Zeng P., Xiao X., Peng C. (2021). Physiological, anatomical, and transcriptional responses of mulberry (*Morus alba* L.) to Cd stress in contaminated soil. Environ. Pollut..

[22] Du Q., Guo P., Shi Y., Zhang J., Zheng D., Li Y., Acheampong A., Wu P., Lin Q., Zhao W. (2021). Genome-wide identification of copper stress-regulated and novel microRNAs in mulberry leaf. Biochem. Genet..

[23] He N., Zhang C., Qi X., Zhao S., Tao Y., Yang G., Lee T.H., Wang X., Cai Q., Li D. (2013). Draft genome sequence of the mulberry tree Morus notabilis. Nat. Commun..

[24] Dhanyalakshmi K.H., Naika M.B., Sajeevan R.S., Mathew O.K., Shafi K.M., Sowdhamini R., Nataraja K.N. (2016). An approach to function annotation for proteins of unknown function (PUFs) in the transcriptome of indian Mulberry. PLoS ONE.

[25] Dhanyalakshmi K.H., Nataraja K.N. (2018). Mulberry (Morus spp.) has the features to treat as a potential perennial model system. Plant Signal. Behav..

[26] Li R., Chen D., Wang T., Wan Y., Li R., Fang R., Wang Y., Hu F., Zhou H., Li L. (2017). High throughput deep degradome sequencing reveals microRNAs and their targets in response to drought stress in mulberry (*Morus alba*). PLoS ONE.

[27] Per T.S., Khan N.A., Reddy P.S., Masood A., Hasanuzzaman M., Khan M., Anjum N.A. (2017). Approaches in modulating proline metabolism in plants for salt and drought stress tolerance: Phytohormones, mineral nutrients and transgenics. Plant Physiol. Biochem..

[28] Zhang X., Liu C.-J. (2015). multifaceted regulations of gateway enzyme phenylalanine ammonia-lyase in the biosynthesis of phenylpropanoids. Mol. Plant.

[29] Vogt T. (2010). Phenylpropanoid biosynthesis. Mol. Plant.

[30] Yang Y., Guo Y. (2018). Elucidating the molecular mechanisms mediating plant salt-stress responses. New Phytol..

[31] Chen Z.H., Chen G., Dai F., Wang Y., Hills A., Ruan Y.L., Zhang G., Franks P.J., Nevo E., Blatt M.R. (2017). Molecular evolution of grass stomata. Trends Plant Sci..

[32] Lawson T., Vialet-Chabrand S. (2019). Speedy stomata, photosynthesis and plant water use efficiency. New Phytol..

[33] Aung K., Jiang Y., He S.Y. (2018). The role of water in plant-microbe interactions. Plant J..

[34] Liu C., Fan W., Zhu P., Xia Z., Hu J., Zhao A. (2019). Mulberry RGS negatively regulates salt stress response and tolerance. Plant. Signal. Behav..

[35] Zhang H., Li X., Guan Y., Li M., Wang Y., An M., Zhang Y., Liu G., Xu N., Sun G. (2020). Physiological and proteomic responses of reactive oxygen species metabolism and antioxidant machinery in mulberry (*Morus alba* L.) seedling leaves to NaCl and NaHCO_3_ stress. Ecotoxicol. Environ. Saf..

[36] Hu H., Xiong L. (2014). Genetic engineering and breeding of drought-resistant crops. Annu. Rev. Plant Biol..

[37] Hong Y., Zhang H., Huang L., Li D., Song F. (2016). Overexpression of a stress-responsive NAC transcription factor gene *ONAC022* improves drought and salt tolerance in rice. Front. Plant Sci..

[38] Thakur M., Sharma A.D. (2005). Salt-stress-induced proline accumulation in germinating embryos: Evidence suggesting a role of proline in seed germination. J. Arid Environ..

[39] Ma J., Du G., Li X., Zhang C., Guo J. (2015). A major locus controlling malondialdehyde content under water stress is associated with *Fusarium* crown rot resistance in wheat. Mol. Genet. Genom..

[40] Morales M., Munné-Bosch S. (2019). Malondialdehyde: Facts and Artifacts. Plant Physiol..

[41] Sandhu D., Cornacchione M.V., Ferreira J.F., Suarez D.L. (2017). Variable salinity responses of 12 alfalfa genotypes and comparative expression analyses of salt-response genes. Sci. Rep..

[42] Zhao W.T., Feng S.J., Li H., Faust F., Kleine T., Li L.N., Yang Z.M. (2017). Salt stress-induced *FERROCHELATASE 1* improves resistance to salt stress by limiting sodium accumulation in *Arabidopsis thaliana*. Sci. Rep..

[43] Lawson T. (2009). Guard cell photosynthesis and stomatal function. New Phytol..

[44] Blatt M.R., Brodribb T.J., Torii K.U. (2017). Small pores with a big impact. Plant Physiol..

[45] Duan H., O’Grady A.P., Duursma R.A., Choat B., Huang G., Smith R.A., Jiang Y., Tissue D.T. (2015). Drought responses of two gymnosperm species with contrasting stomatal regulation strategies under elevated [CO_2_] and temperature. Tree Physiol..

[46] Urban J., Ingwers M., McGuire M.A., Teskey R.O. (2017). Stomatal conductance increases with rising temperature. Plant Signal. Behav..

[47] Eller F., Lambertini C., Nguyen L.X., Brix H. (2014). Increased invasive potential of non-native *Phragmites australis*: Elevated CO_2_ and temperature alleviate salinity effects on photosynthesis and growth. Glob. Chang. Biol..

[48] Kumar S.G., Madhusudhan K.V., Sreenivasulu N., Sudhakar C. (2000). Stress responses in two genotypes of mulberry (*Morus alba* L.) under NaCl salinity. Ind. J. Exp. Biol..

[49] Pérez-López U., Robredo A., Lacuesta M., Mena-Petite A., Muñoz-Rueda A. (2012). Elevated CO2 reduces stomatal and metabolic limitations on photosynthesis caused by salinity in *Hordeum vulgare*. Photosynth. Res..

[50] Luo M., Zhao Y., Wang Y., Shi Z., Zhang P., Zhang Y., Song W., Zhao J. (2018). Comparative proteomics of contrasting maize genotypes provides insights into salt-stress tolerance mechanisms. J. Proteome Res..

[51] Meng X., Liu S., Dong T., Xu T., Zhu M. (2020). Comparative transcriptome and proteome analysis of salt-tolerant and salt-sensitive sweet potato and overexpression of IbNAC7 confers salt tolerance in Arabidopsis. Front. Plant Sci..

[52] Baâtour O., Mahmoudi H., Tarchoun I., Nasri N., Trabelsi N., Kaddour R., Zaghdoudi M., Hamdawi G., Ksouri R., Lachaâl M. (2013). Salt effect on phenolics and antioxidant activities of *Tunisian* and *Canadian sweet marjoram* (*Origanum majorana* L.) shoots. J. Sci. Food Agric..

[53] Chen F., Fang P., Peng Y., Zeng W., Ren B. (2019). Comparative proteomics of salt-tolerant and salt-sensitive maize inbred lines to reveal the molecular mechanism of salt tolerance. Int. J. Mol. Sci..

[54] Dong N.Q., Lin H.X. (2021). Contribution of phenylpropanoid metabolism to plant development and plant-environment interactions. J. Integr. Plant Biol..

[55] Silva R.R.D., Câmara C.A.G.D., Almeida A.V., Ramos C.S. (2012). Biotic and abiotic stress-induced phenylpropanoids in leaves of the mango (*Mangifera indica* L., Anacardiaceae). J. Braz. Chem. Soc..

[56] Falcone Ferreyra M.L., Rodriguez E., Casas M.I., Labadie G., Grotewold E., Casati P. (2013). Identification of a bifunctional maize C- and O-Glucosyltransferase. J. Biol. Chem..

[57] Casas M.I., Ferreyra M.L.F., Jiang N., Mejía-Guerra M.K., Rodríguez E., Wilson T., Engelmeier J., Casati P., Grotewold E. (2016). Identification and characterization of maize salmon silks genes involved in insecticidal maysin biosynthesis. Plant Cell.

[58] Russell W.R., Burkitt M.J., Scobbie L., Chesson A. (2006). EPR investigation into the effects of substrate structure on peroxidase-catalyzed phenylpropanoid oxidation. Biomacromolecules.

[59] Lai Y., Zhang D., Wang J., Wang J., Ren P., Yao L., Si E., Kong Y., Wang H. (2020). Integrative transcriptomic and proteomic analyses of molecular mechanism responding to salt stress during seed germination in Hulless Barley. Int. J. Mol. Sci..

[60] Geng G., Lv C., Stevanato P., Li R., Liu H., Yu L., Wang Y. (2019). Transcriptome analysis of salt-sensitive and tolerant genotypes reveals salt-tolerance metabolic pathways in sugar beet. Int. J. Mol. Sci..

[61] Zheng L., Meng Y., Ma J., Zhao X., Cheng T., Ji J., Chang E., Meng C., Deng N., Chen L. (2015). Transcriptomic analysis reveals importance of ROS and phytohormones in response to short-term salinity stress in *Populus tomentosa*. Front. Plant Sci..

[62] Liu C., Xu Y., Feng Y., Long D., Cao B., Xiang Z., Zhao A. (2018). Ectopic expression of mulberry G-proteins alters drought and salt stress tolerance in Tobacco. Int. J. Mol. Sci..

