# Supplementary figures and images for: Comparative Proteomic Analysis of Tolerant and Sensitive Varieties Reveals That Phenylpropanoid Biosynthesis Contributes to Salt Tolerance in Mulberry
Source: Int J Mol Sci. 2021 Aug 30;22(17):9402. doi: 10.3390/ijms22179402 (PMC8431035; doi:10.3390/ijms22179402)

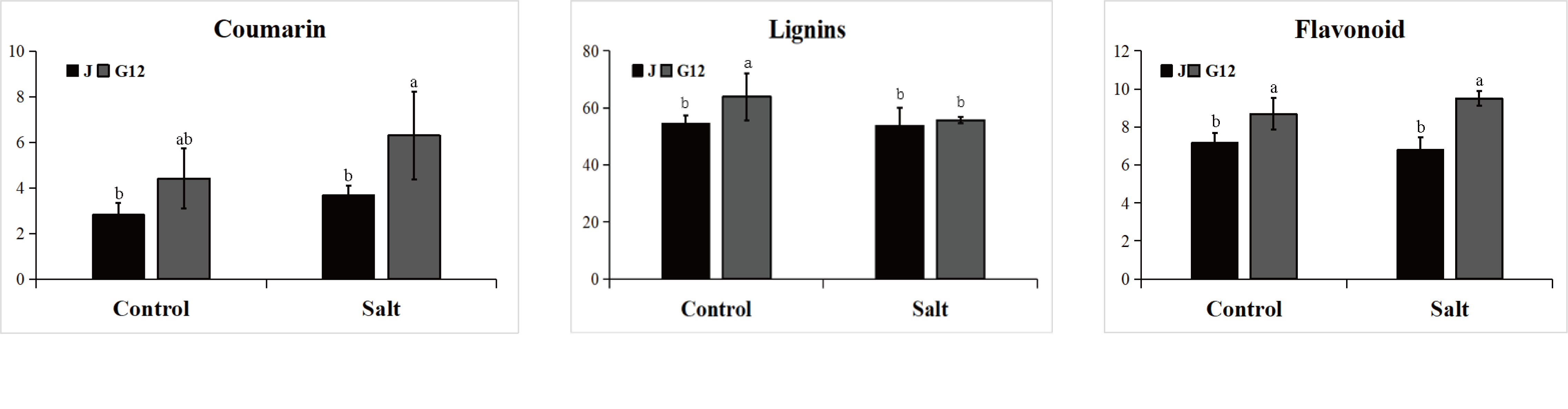

Supplement: Supplementary file 1 [file ijms-22-09402-s001.zip › Supplementary Files/Figure S7 .tif]

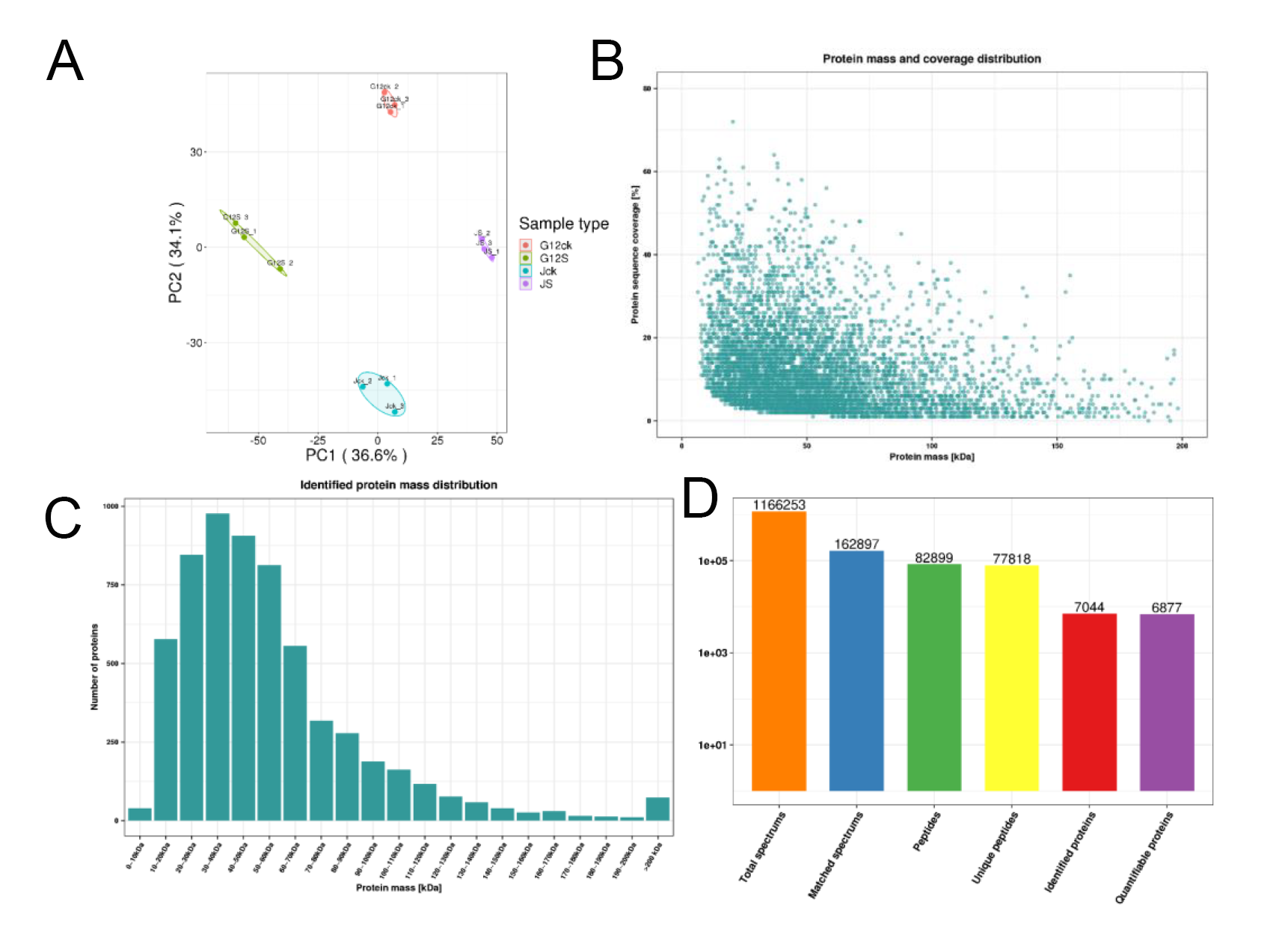

Supplement: Supplementary file 1 [file ijms-22-09402-s001.zip › Supplementary Files/figure S1.tif]

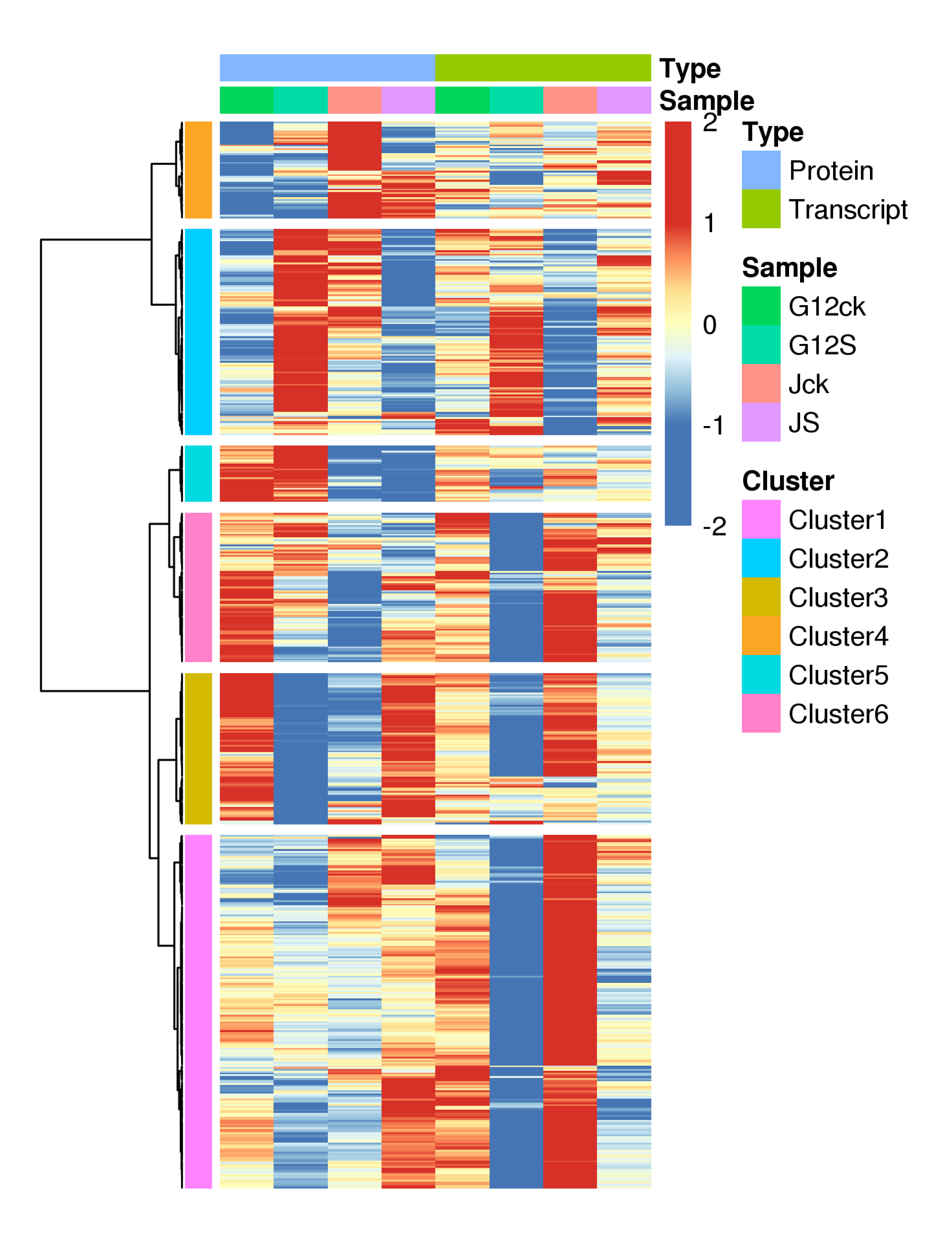

Supplement: Supplementary file 1 [file ijms-22-09402-s001.zip › Supplementary Files/figure S10.tif]

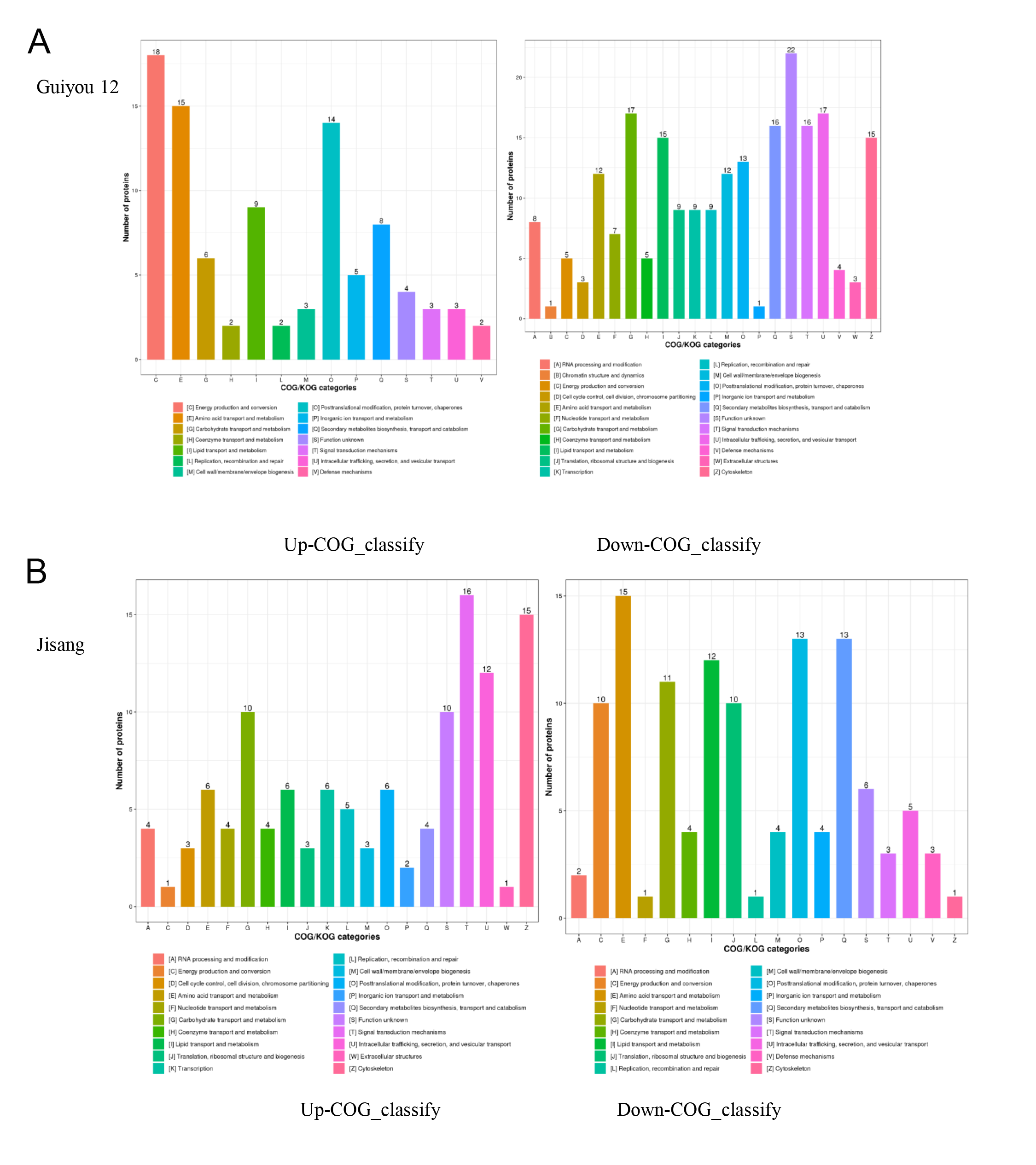

Supplement: Supplementary file 1 [file ijms-22-09402-s001.zip › Supplementary Files/figure S2.tif]

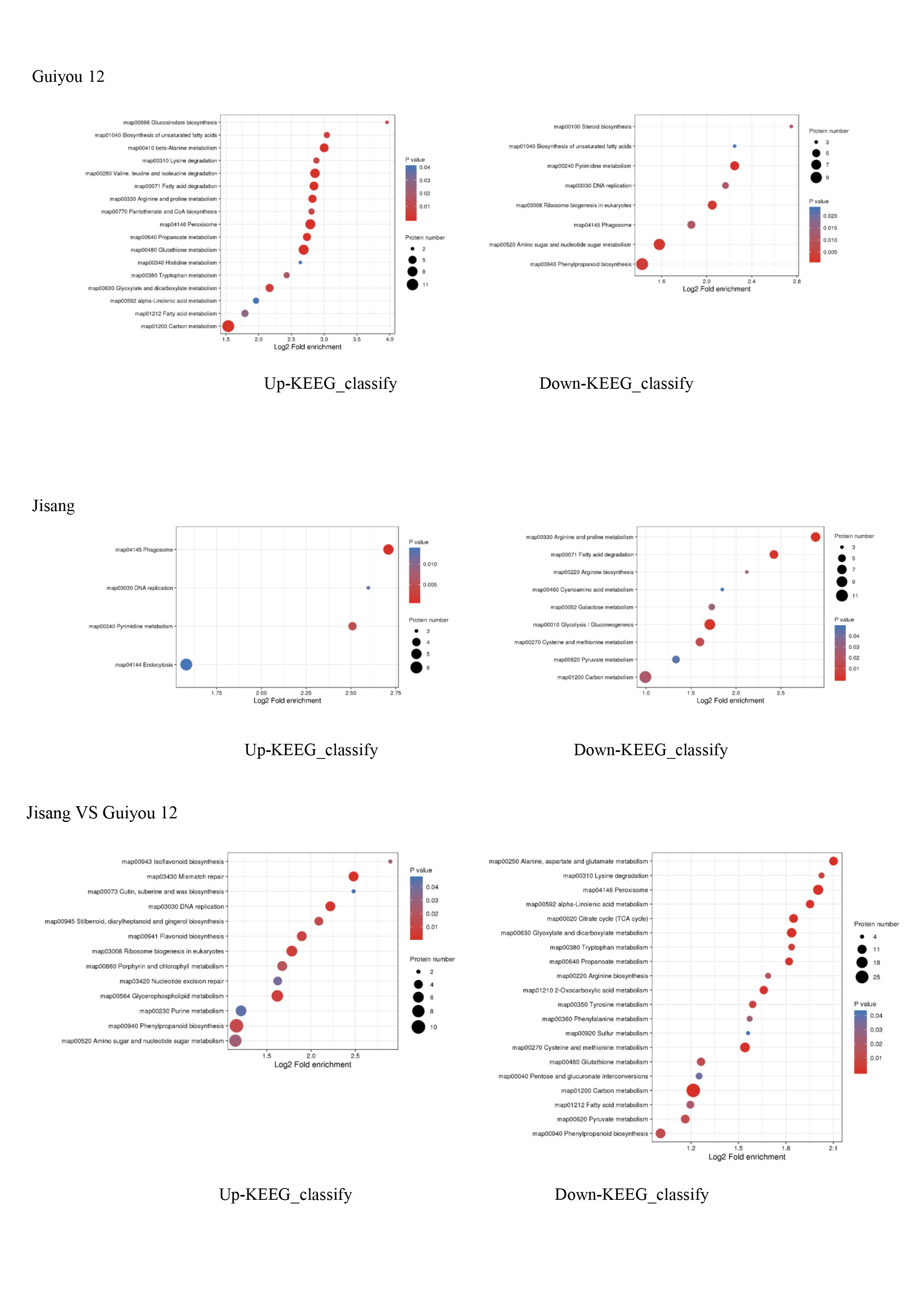

Supplement: Supplementary file 1 [file ijms-22-09402-s001.zip › Supplementary Files/figure S3.tif]

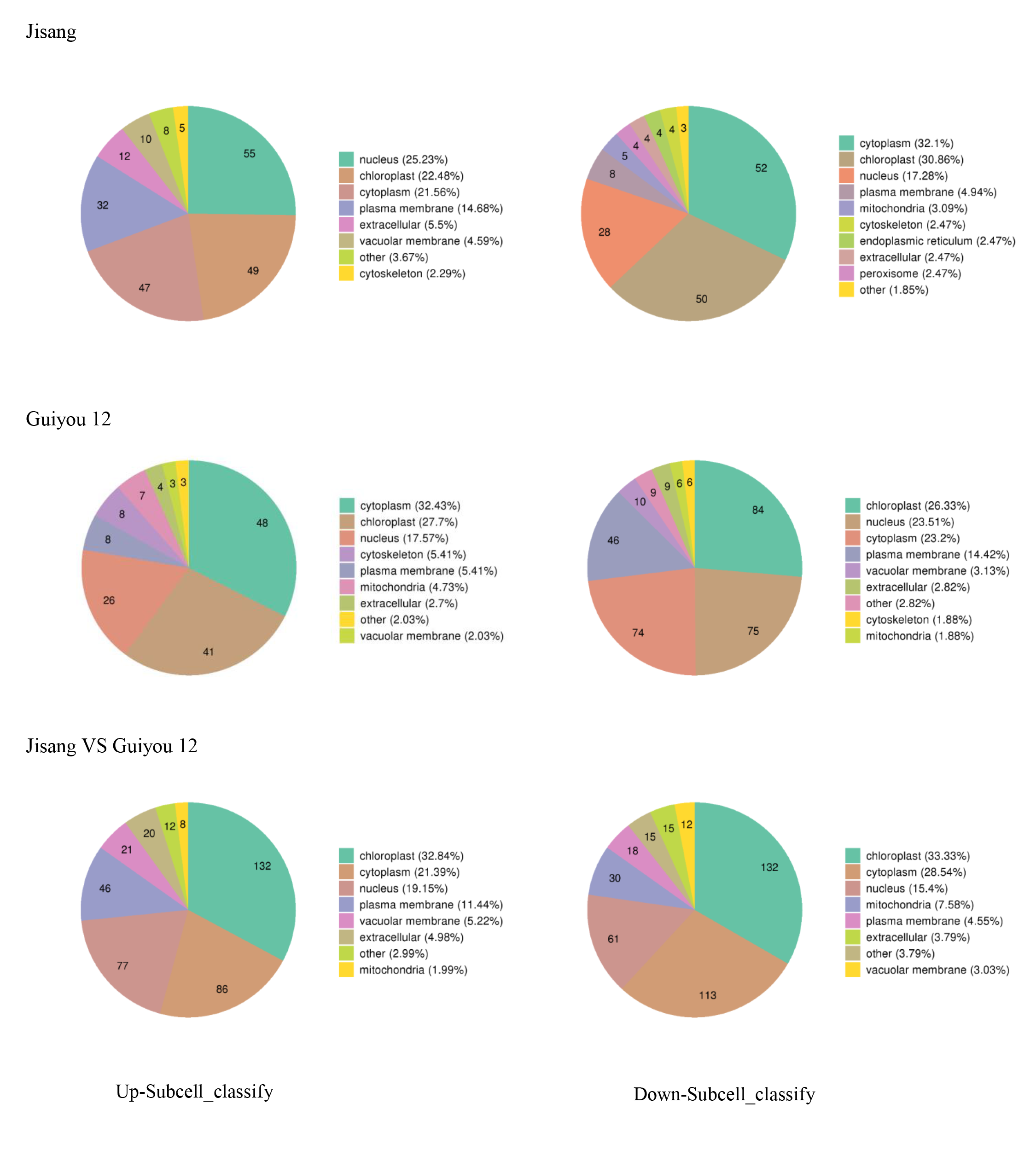

Supplement: Supplementary file 1 [file ijms-22-09402-s001.zip › Supplementary Files/figure S4.tif]

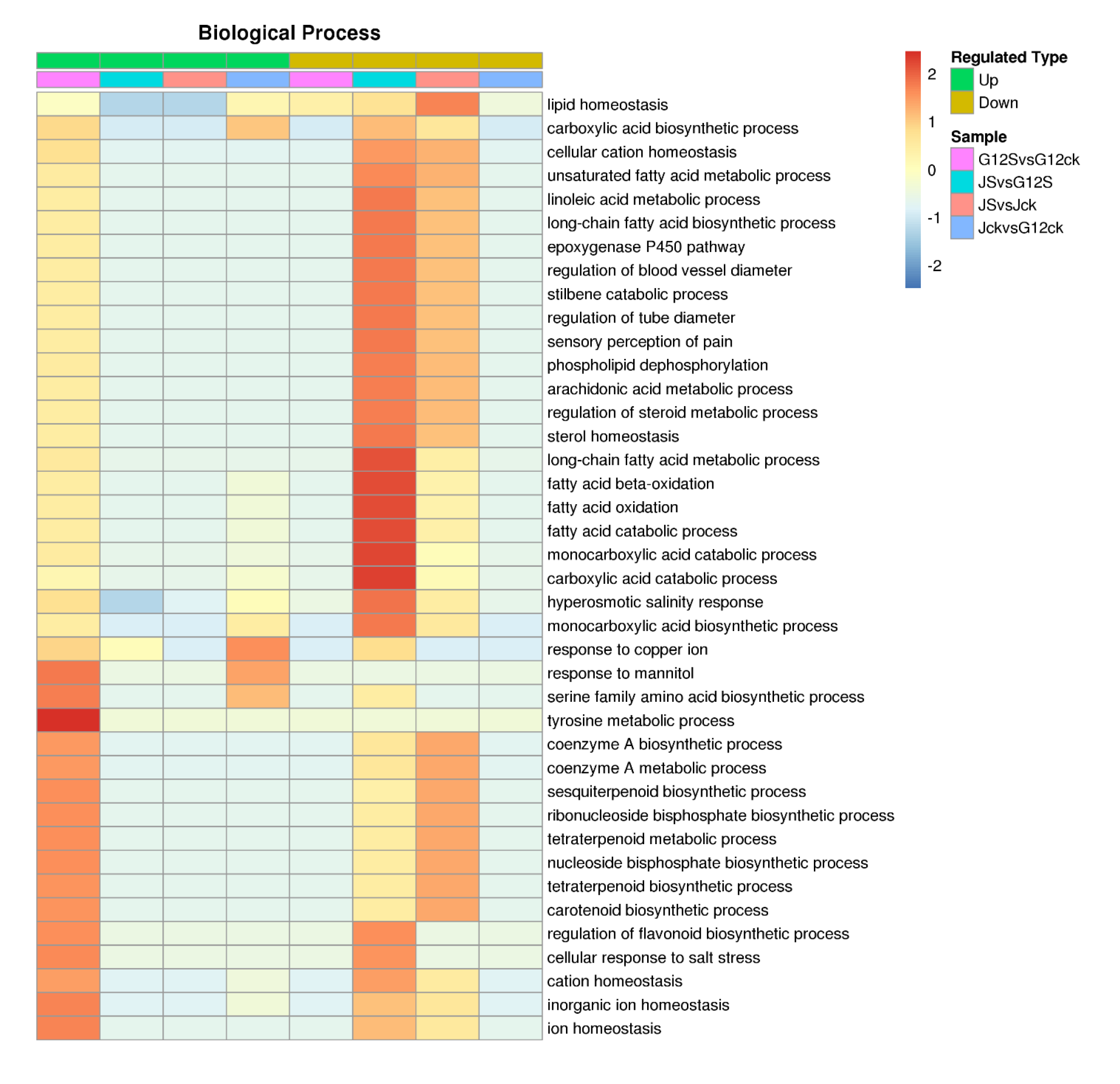

Supplement: Supplementary file 1 [file ijms-22-09402-s001.zip › Supplementary Files/figure S5.tif]

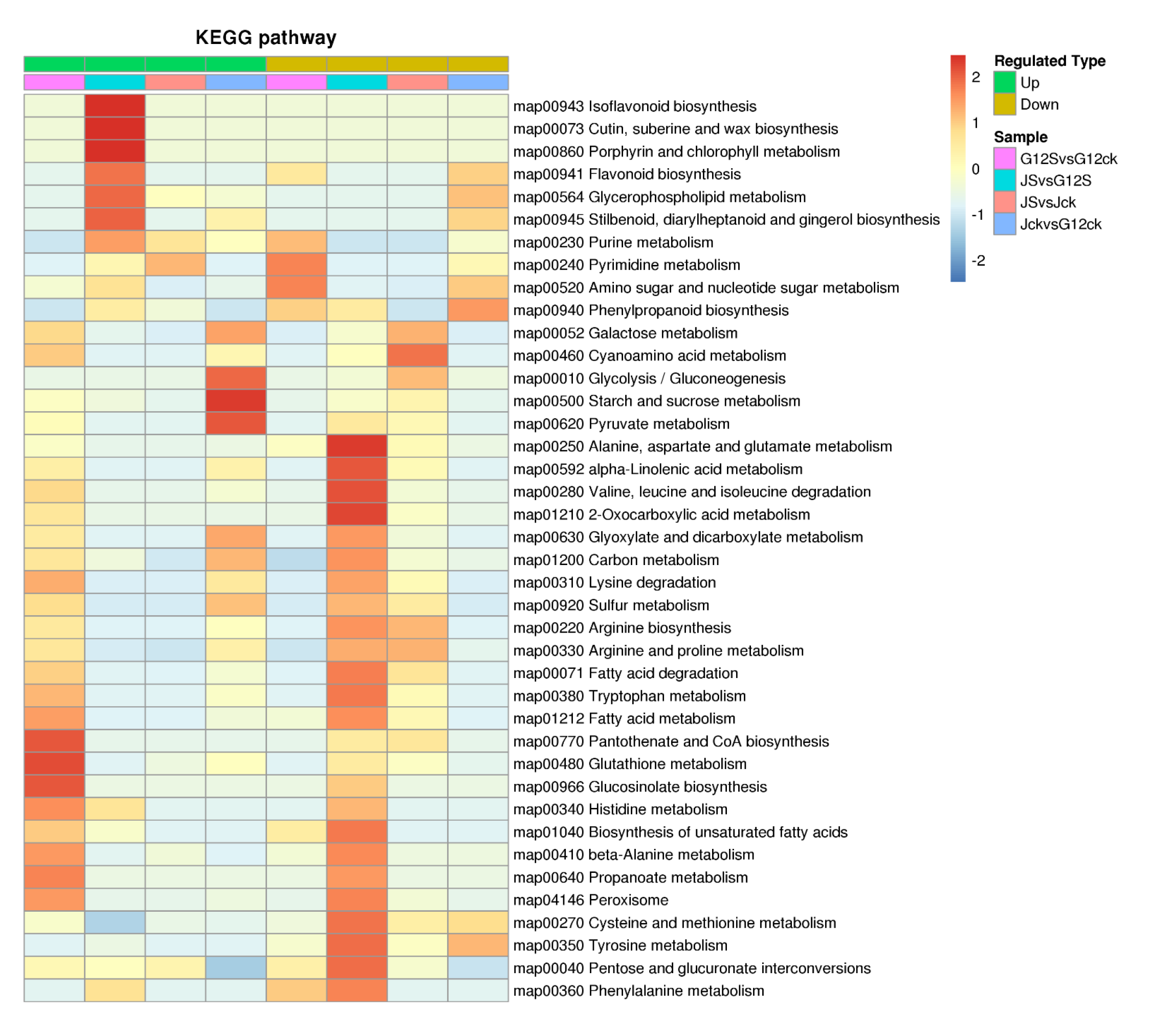

Supplement: Supplementary file 1 [file ijms-22-09402-s001.zip › Supplementary Files/figure S6.tif]

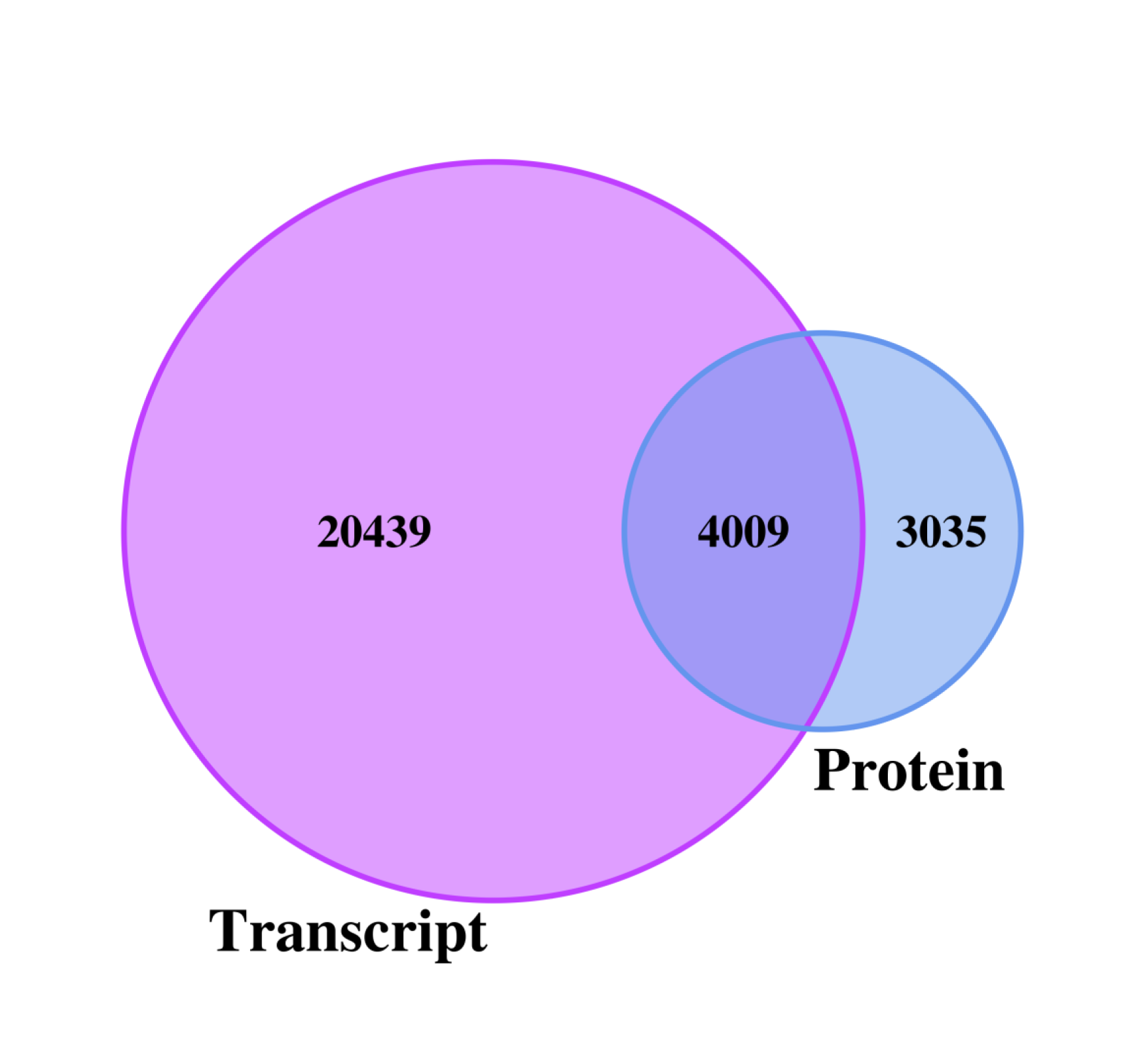

Supplement: Supplementary file 1 [file ijms-22-09402-s001.zip › Supplementary Files/figure S8.tif]

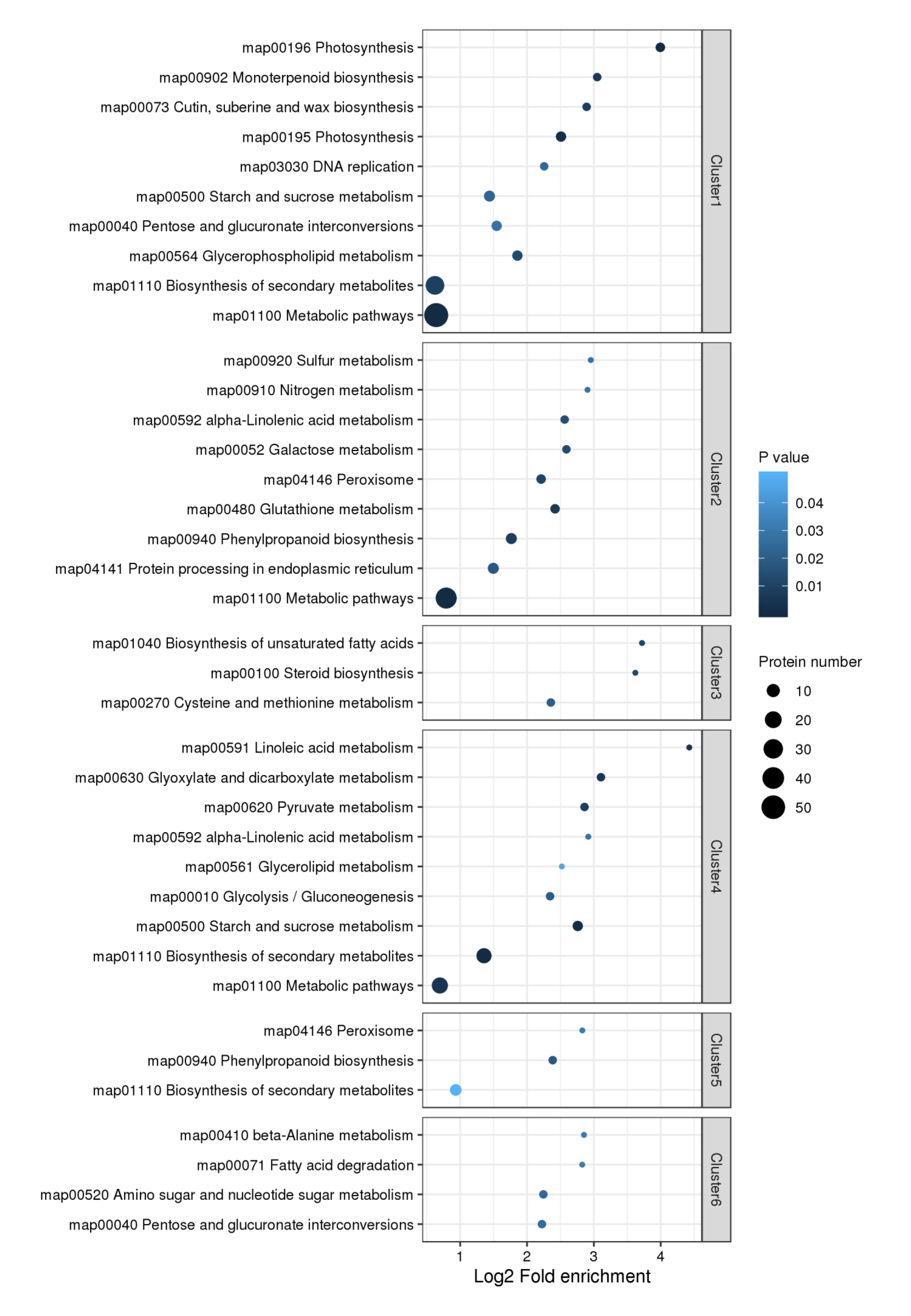

Supplement: Supplementary file 1 [file ijms-22-09402-s001.zip › Supplementary Files/figure S9.tif]
